# Social anxiety under load: the effects of perceptual load in processing emotional faces

**DOI:** 10.3389/fpsyg.2015.00479

**Published:** 2015-04-21

**Authors:** Sandra C. Soares, Marta Rocha, Tiago Neiva, Paulo Rodrigues, Carlos F. Silva

**Affiliations:** ^1^Center for Health Technology and Services Research (CINTESIS), Department of Education, University of Aveiro, Aveiro, Portugal; ^2^Department of Education, University of Aveiro, Aveiro, Portugal; ^3^Department of Psychology and Education, The University of Beira Interior, Covilhã, Portugal

**Keywords:** attentional control, emotional faces, social anxiety, perceptual load, target discrimination task

## Abstract

Previous studies in the social anxiety arena have shown an impaired attentional control system, similar to that found in trait anxiety. However, the effect of task demands on social anxiety in socially threatening stimuli, such as angry faces, remains unseen. In the present study, 54 university students scoring high and low in the Social Interaction and Performance Anxiety and Avoidance Scale (SIPAAS) questionnaire, participated in a target letter discrimination task while task-irrelevant face stimuli (angry, disgust, happy, and neutral) were simultaneously presented. The results showed that high (compared to low) socially anxious individuals were more prone to distraction by task-irrelevant stimuli, particularly under high perceptual load conditions. More importantly, for such individuals, the accuracy proportions for angry faces significantly differed between the low and high perceptual load conditions, which is discussed in light of current evolutionary models of social anxiety.

## Introduction

Emotion and attention share important evolutionary-driven functions and are interconnected in the sense that they both deal with information processing priorities (Oatley and Johnson-Laird, 1987). Threatening stimuli that are deeply rooted in evolution, such as angry faces (for reviews, see Öhman, 2009; Öhman et al., 2012), seem to have a unique status in such interactions. Such face threat laden stimuli engage evolutionary shaped behavior systems and show more persistent conditioned fear responding (e.g., Öhman et al., 1985), heightened psychophysiological responses (e.g., Öhman and Soares, 1994), and attentional priority in visual search settings (e.g., Öhman et al., 2010).

Individuals with social anxiety disorder (SAD), who are highly fear of negative social interactions (Diagnostic and Statistical Manual of Mental Disorders, DSM-V; American Psychiatric Association [APA], 2013), are particularly sensitive to threat in faces, as a result of their increased sensitivity for facial signals of social dominance (e.g., Öhman, 1986). There is mounting evidence from a wide range of tasks and methodologies showing that both clinical and non-clinical individuals with social anxiety show a cognitive bias in processing social information, such as an enhanced automatic processing of angry faces (for reviews, see Bar-Haim et al., 2007; Staugaard, 2010; Morrison and Heimberg, 2013; Schulz et al., 2013). In fact, the central tenet of several models of anxiety is to attribute the etiology and maintenance of SAD to the automatic processing of social threat signals in faces (for a review, see Van Bockstaele et al., 2014). For instance, according to one of the dominant cognitive models of SAD, proposed by Rapee and Heimberg (1997), socially anxious individuals automatically direct their attention to potentially threatening stimuli, such as negative faces, which then serves to maintain their anxiety since they confirm their social fears by the elaborate processing of the fear signals in the environment.

Automatic information processing is assumed to be fast, involuntary and non-strategical, as opposed to goal-directed behavior, which is dependent on top-down attentional control (e.g., Yantis, 1993). The demands of the task determine the extent to which we employ attentional control, i.e., attend goal-relevant stimuli at the expense of irrelevant stimuli (e.g., Lavie et al., 2004). According to the attentional control theory (ACT; Eysenck et al., 2007), performance in demanding tasks should be hinder in individuals with deficits in attentional control, as it is the case with anxious individuals. Indeed, the optimal strategy for anxious individuals is to allocate more attentional resources to process a great amount of information, as this increases the likelihood of detecting threat-related information (e.g., Öhman, 1986; Rapee and Heimberg, 1997; Williams et al., 1997; Mogg and Bradley, 1998). Such strategy results in a lower efficiency in inhibiting task-irrelevant stimuli (Berggren and Derakshan, 2013). However, although the ACT predicts that anxious individuals, compared to low anxious people, allocate more attentional resources to overcome their attentional control deficits, the theory does not directly address if or how deficits in attentional control in anxiety are dependent on the perceptual load of the task at hand. Instead, the cognitive demands have been assessed by including a secondary task or by increasing the amount of information in working memory tasks (e.g., Moriya and Sugiura, 2012).

According to the work developed by Lavie (1995, 2005), stimuli that are task-irrelevant (distractors) are not processed beyond a fairly superficial level when perceptual resources are fully occupied in an ongoing task. In contrast, when the perceptual load involved in the task is low, and perceptual capacities involved in the task are not exhausted, there are more resources available for processing the distractors. Accordingly, previous studies have showed that at high perceptual load, processing emotional facial information unrelated to the task is prevented (e.g., Pessoa et al., 2005; Bishop et al., 2007; Lim et al., 2008). However, a different set of studies have shown the opposite pattern of results, i.e., face distractors are prioritized irrespective of the perceptual load of the task (e.g., Vuilleumier et al., 2001), with the underlying interpretation of the data being based on the biological significance of the face stimuli (e.g., Öhman et al., 2012). In fact, cognitive models of anxiety predict a mandatory process of threatening stimuli in anxious individuals, even under high task demands (Eysenck et al., 2007).

Although several studies have indicated an impaired attentional control in anxious individuals (e.g., Berggren and Derakshan, 2013), research investigating if the processing of task-irrelevant stimuli is affected by the task demands is scant, particularly in the social anxiety domain. A recent study with SAD patients (compared to controls) showed that specific differences in brain responses to threatening facial stimuli were relatively immune to task implicit or explicit task requirements (designed to manipulate the focus of attention). More importantly, such differences were more pronounced when the face stimuli were task-irrelevant (Straube et al., 2011). However, this study did not investigate the efficiency of processing task-irrelevant stimuli, which could be done by varying the perceptual load imposed by the main task (e.g., Lavie, 1995, 2005). In line with this, a few studies have investigated whether efficient attention to task-irrelevant stimuli was maintained regardless of cognitive resources in individuals with social anxiety (Moriya and Tanno, 2010, 2011; Moriya and Sugiura, 2012). The results showed that high social anxious (HSA) individuals did not inhibit the processing of task-irrelevant stimuli under high perceptual load conditions, as would be predicted by the perceptual load theory (Lavie, 1995). However, these studies only included non-emotional stimuli (e.g., letters). Thus, it remains unseen whether socially threatening stimuli, such as facial stimuli, which are particularly significant in SAD (for a review, see Staugaard, 2010), would disrupt the enhanced processing of task-irrelevant stimuli.

In the present study the purpose was to test whether processing task-irrelevant emotional stimuli is enhanced in HSA individuals (compared to low anxious individuals), even when perceptual resources are fully engaged in a highly demanding primary task (Forster and Lavie, 2008). Moreover, we aimed at investigating if such interference effects of task-irrelevant stimuli are restricted to threatening faces, compared to positive and neutral ones.

## Materials and Methods

### Participants

Fifty-four students (26 men and 28 women) from the University of Aveiro, Portugal, aged between 18 and 56 years (*M* = 22.06; SD = 5.58), volunteered to participate. Participants were divided into high and low levels of anxiety and avoidance in social situations by using the Social Interaction and Performance Anxiety and Avoidance Scale (SIPAAS; Pinto-Gouveia et al., 2003). This scale comprises 44 items that represent performance and social interaction situations (e.g., “*Go to a party*”, “*Ask someone out*”, “*Do an oral exam*”, “*Ask a stranger for information*”). For each situation, participants are asked to rate in a 4-point Likert scale (1–4) the degree of discomfort/anxiety felt and the extent to which they avoid that situation. This measure includes two subscales: Discomfort/Anxiety and Avoidance, although we only used the total score in the present study, which ranged from 88 to 352.

Twenty-seven participants were allocated in a HSA group (*M* = 206.74; SD = 32.02) and 27 participants in a low social anxious (LSA) group (*M* = 136.85; SD = 17.20). We used the median split to divide the participants into the HSA and LSA groups, with the scores in SIPAAS showing statistically significant differences, *t*(52) = 9.99, *p* < 0.001. Importantly, this procedure allowed for a direct comparison between our results and those by Moriya and Tanno (2010), which we wanted to extend in the present study. Participants also completed a Portuguese version of the Beck Depression Inventory (Beck et al., 1961). However, no significant differences were shown between groups regarding their levels of depression, *t*(52) = 1.06, *p* > 0.05. The selection procedure to create the HSA group resulted only in participants with high levels of anxiety and avoidance of social situations, but did not include a formal DSM-V (APA, 2013) diagnosis of SAD. The participants had normal or corrected-to-normal vision, did not suffer from any mental or neurological illness and were medication free. Participants in both groups were matched for age, gender, and handedness.

The study was approved by the Ethics Committee of University of Aveiro, Portugal and the guidelines of the Declaration of Helsinki. Moreover, standards of American Psychological Association were followed. Participation as subjects in the experiment was based on written informed consent including the right to abort participation at any time. Participants were rewarded with course credits.

### Equipment and Materials

All stimuli were presented against a white background. Target stimuli consisted of a character (X or N). Non-target letters were randomly chosen from G, H, K, J, S, Y. Participants were seated 40 cm from the screen. Target stimuli were presented around an imaginary circle, with a 2.52° radius. All letters were presented in font type “Lucida Console,” and were 0.5° in width and 0.5° in height.

The task-irrelevant stimuli consisted of four male and four female faces, selected from the Karolinska Directed Emotional Faces (identity-numbers AF01, AF09, AF22, AF26, AM08, AM10; AM17, AM29; Lundqvist et al., 1998, http://www.facialstimuli.com/). Each individual displayed each of the following emotional categories: negative (angry and disgust), positive (happy), and neutral. The size of each face (i.e., task-irrelevant distractor picture) was 6.45° in width by 6.46° in height, and were displayed 9.45° from fixation to the center of the picture.

The task was programmed using the software E-prime 2.0 (Schneider et al., 2002) and the stimulus presentation was conducted using a Dell OptiPlex 745 and an LG Flatron W2246 monitor with a 22-inch monitor. The monitor had a refresh rate of 60 Hz. Participants used the letters X and N on the keyboard for their responses.

### Task and Procedure

**FIGURE 1:**
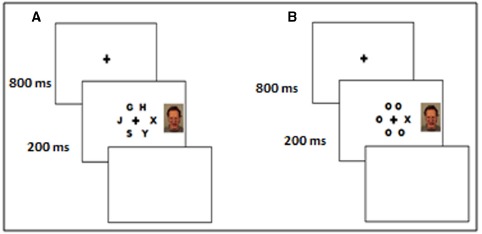
**Sequence of the high (A) and low (B) perceptual load conditions in the experiment**.

After giving informed consent, participants were asked to find a position in the chair where they could comfortably reach the two response keys with their right and left index fingers. We used a central task with brief stimulus duration (200 ms), where participants had to decide, as quickly and accurately as possible, the identity a designated target letter (X or N) presented at the center of the display. The target letter was presented among five “Os” (low perceptual load) on 50% of the total number of trials or surrounded by five non-target letters—G, H, J, S, Y (high perceptual load), arranged in a circular display (see Figure 1). The position of the target letter surrounded fixation on every trial and its presentation was randomized over the six possible positions.

Each trial started with the presentation of a black fixation cross against a white background. The fixation cross was randomly presented for 800 or 1200 ms, and immediately followed by the stimulus display, after which a blank white response screen was presented until the participants response. The intertrial interval was 500 ms. On each trial, a task-irrelevant distractor image depicting an angry, disgust, happy, or neutral face, was displayed either to the left or to the right of fixation (equal probability). The face stimulus was presented simultaneously with the stimulus display (Figure 1). The order of the perceptual load level and stimuli (image distractor type, target letter, non-target letters) was fully randomized for each participant (see Moriya and Tanno, 2011). Participants initially completed 64 practice trials (16 for each facial expression, equally distributed by the perceptual load level) with accuracy feedback. None of the faces used in the practice was shown in the main experiment. Practice trials were followed by two blocks of experimental trials. Each block consisted of 288 trials, 144 for low and 144 for high load conditions (36 for each face type: angry, disgust, happy, and neutral, in both low and high load conditions). A participant-paced pause interval occurred between each block.

### Design and Statistical Analyses

The analysis of response times (RTs) excluded error trials and outliers were removed by plotting the individual data points (4.12%). Separate analyses were calculated for correct RT (ms) and for proportion of response accuracy (%). Follow-up tests were accomplished using Tukey’s HSDs (honest significant differences). Significance levels were set at *p* < 0.05, and partial η^2^ (${\text{η}}_{\text{p}}^{\text{2}}$) were used as estimate of effect sizes.

A mixed effects repeated measures ANOVA was conducted for both RT and accuracy, with perceptual load (low/high) and distractor (neutral, happy, angry, disgust) as within-participants factors, and social anxiety (low/high) as between participants factor.

## Results

### Response Times

**FIGURE 2:**
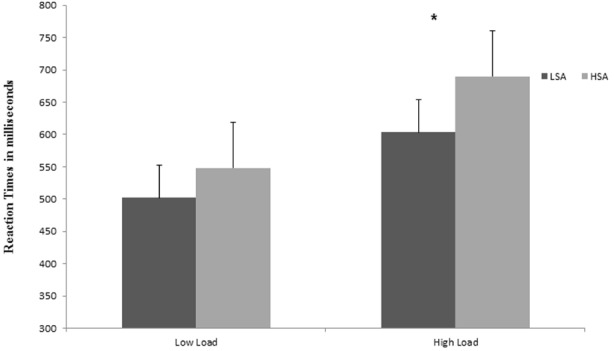
**Mean reaction times (RTs) in milliseconds (ms) to discriminate the target letter (X or N) in the different perceptual load conditions (low and high), as a function of the group (LSA, low social anxiety; HSA, high social anxiety).** Longer RTs indicate larger interference scores. *p < 0.05.

The results showed a very robust main effect of perceptual load, with slower overall RTs at high load (*M* = 647 ms; SD = 135 ms) than at low load conditions (*M* = 525 ms; SD = 87 ms), *F*(1,52) = 205.48, *p* < 0.0001, ${\text{η}}_{\text{p}}^{\text{2}}$ = 0.80, thus confirming the effectiveness of the perceptual load manipulation. The results also revealed a two-way interaction between social anxiety and perceptual load, *F*(1,52) = 5.62, *p* < 0.05, ${\text{η}}_{\text{p}}^{\text{2}}$ = 0.10, showing that only the HSA, in contrast with the LSA, had statistically significantly longer RTs under the high load conditions (Tukey’s HSD, *p* < 0.05), compared to the low load (Tukey’s HSD, *p* = 0.40; Figure 2).

Finally, there was a main effect of social anxiety, showing that RTs were significantly slower in the HSA group (*M* = 619 ms; SD = 136 ms), compared to the LSA group (*M* = 553 ms; SD = 111 ms;), *F*(1,52) = 5.57, *p* < 0.05, ${\text{η}}_{\text{p}}^{\text{2}}$ = 0.10. No other significant main effects or interactions were found.

### Accuracy

**FIGURE 3:**
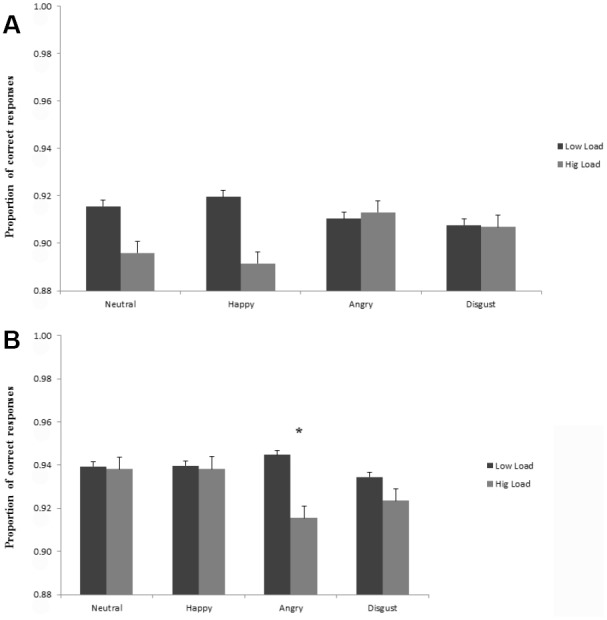
**(A)** Mean accuracy proportions for the low social anxious to discriminate the target letter (X or N) in the different perceptual load conditions (low and high), as a function of the face distractor (neutral, happy, angry, disgust). Lower accuracy proportions indicate larger interference scores. **(B)** Mean accuracy proportions for the high social anxious to discriminate the target letter (X or N) in the different perceptual load conditions (low and high), as a function of the face distractor (neutral, happy, angry, disgust). Lower accuracy proportions indicate larger interference scores.

The analysis of accuracy showed a main effect of perceptual load, analogous to the effect in RTs, *F*(1,52) = 5.85, *p* < 0.05, ${\text{η}}_{\text{p}}^{\text{2}}$ = 0.10, with overall lower accuracy proportions at high load (*M* = 0.92; SD = 0.07), compared to low load conditions (*M* = 0.93; SD = 0.07). Moreover, the results revealed a three-way interaction between social anxiety, face distraction and perceptual load, *F*(3,156) = 4.97, *p* < 0.01, ${\text{η}}_{\text{p}}^{\text{2}}$ = 0.09. HSA individuals, compared LSA, showed higher accuracy in the main task (discriminate the target letter), in the high perceptual load conditions, consistently with the RT results. Moreover, while for LSA individuals no differences in accuracy were shown for the different emotional faces both in the low and high load conditions (Figure 3A), in the HSA group, there was a statistically significant difference in the *post hoc* Tukey’s tests for angry faces between the low and high load conditions, showing a lower accuracy for angry faces in the high load conditions (Tukey’s HSDs, *p* < 0.05; see Figure 3B).

No main effects of group (*p* = 0.90), distractor type (*p* = 0.83), and no perceptual load by distractor type (*p* = 0.74), perceptual load by group (*p* = 0.94), and distractor type by group (*p* = 0.44) interactions were shown.

## Discussion

Although some studies have investigated the effects of perceptual load in attentional control in trait anxious individuals (see Berggren and Derakshan, 2013 for a review), far less research has investigated such effects in social anxiety. Moreover, those few studies only included non-emotional stimuli, such as letters or natural scenes (Moriya and Tanno, 2010, 2011). In the present study, we used emotional face stimuli, which are highly relevant to social anxiety since they represent potent social cues and provide important and direct feedback in social interactions (for a review, see Staugaard, 2010). The aim of this study was to examine the influence of emotional face task-irrelevant stimuli on top-down attentional control processes in HSA, in comparison with LSA, by using a target letter discrimination task (Forster and Lavie, 2008).

The results showed that, in general, HSA participants’ slowed RT performance independently of the type of face stimuli valence. This effect confirms previous findings with non-emotional stimuli showing that HSA are overall more prone to distraction by task-irrelevant stimuli, independently of the perceptual demands of the task (Moriya and Tanno, 2010, 2011). Importantly, this effect was more pronounced under high perceptual load conditions, thus pointing to an attentional control deficit in HSA participants, which mirror that observed with trait anxiety (e.g., Eysenck et al., 2007).

An additional interpretation of the increased RTs may involve the fact that high HSA participants may have invested more effort to solve the task (regardless of the type of distractors), thus leading to higher accuracy rates. In fact, and concurrently with the RTs, the accuracy results also showed that HSA were overall more accurate in the main task than LSA participants, although the main effect of group was not statistically significant. Interestingly, however, differential effects of face type for HSA, compared to LSA, were shown under high load conditions but not in low load conditions. More specifically, for HSA, angry faces, compared to the other emotional face categories, resulted in a higher interference with the task (lower accuracy) under the high load conditions.

Previous studies have shown that anxiety results in poorer efficiency than effectiveness, i.e., anxious individuals are able to show high levels of performance, compared to low anxious individuals, although this is achieved at the cost of an increased effort to bolster deficits in attentional control (Eysenck and Calvo, 1992; Berggren and Derakshan, 2013). Our study adds the notion that both efficiency and effectiveness are hampered, particularly in socially anxious and when the task-irrelevant stimuli display a threatening content (angry or disgust faces, although the only statistically significant effect was observed for angry faces). Although this is effect may be in part due to the higher performance in the high anxiety group during the low load condition, it was specifically observed in the HSA group, who seemed to have been able to efficiently ignore the distractors under the low perceptual load conditions.

Angry faces represent a form of human hostility of the beholder and, therefore, have served as an important threat signal throughout evolution (see Öhman, 2009; Öhman et al., 2012 for a review). Thus, based on its deep evolutionary origin, they are processed rapidly and efficiently with only minimal analysis of the stimulus input (LeDoux, 2000). In addition, the social fear systems are at the core of SAD (APA, 2013), with a potentiated automatic attentional orienting to angry threat as a natural result in social anxious individuals (see the meta-analysis by Bar-Haim et al., 2007). Congruently, our study provided indications that the hampered effectiveness for angry faces was only observed in individuals prone to display social anxiety and avoidance of social interactions, i.e., with a pre-existing attentional bias to threat in faces. Thus, perceptual load (or task demands) seemed to influence the compensatory strategies used by social anxious individuals to overcome their attentional control deficits. Indeed, several studies have found empirical evidence for the notion that anxiety interferes with top-down attentional control (Bishop et al., 2004, 2007; Bishop, 2007).

One of the central features of cognitive models of anxiety, such as the one proposed by Rapee and Heimberg (1997), is the automatic nature of processing disorder-related stimuli (Bar-Haim et al., 2007), even in conditions where perceptual resources are depleted and would not be appropriate to pursue a deep cognitive analysis (LeDoux, 2000). Hence, the results from the present study showed that even under exhausted cognitive resources, threat biases in social anxiety seemed to operate at early stages of information processing (200 ms; e.g., Mogg et al., 2004; Miskovic and Schmidt, 2012). Such threat bias to angry faces inhibited the performance in the main task under high load conditions, thus resulting in lower accuracy in letter discrimination when angry faces were presented as task-irrelevant stimuli. This reflects that the strategies to compensate the attentional control deficits in HSA did not seem to effectively cover angry faces under load, when additional attentional resources needed to be recruited, contrary to several pervasive findings with trait anxious individuals (e.g., Berggren et al., 2013; but see Ladouceur et al., 2009).

Finally, and in contrast with a wealth of research, our results did not show a threat bias at low load conditions in HSA (for similar findings, see, e.g., Berggren et al., 2013). This result is consistent with the ACT (Eysenck et al., 2007), which predicts that at low load anxious individuals tend to consume additional attentional resources to compensate for attentional control deficits, thus leaving no differential effects between emotions (e.g., Sadeh and Bredemeier, 2011). The processing of emotional relevant stimuli (e.g., snakes and angry faces) is known to be prioritized independently of the attentional conditions, such as the foveal or peripheral presentation in the visual field, as it enables safe avoidance or escape (e.g., Öhman et al., 2012; Soares et al., 2014), which motivated the peripheral presentation of the task-irrelevant stimuli in the peripheral visual field. However, studies have also showed that face stimuli are preferentially processed in central vision (e.g., Holmes et al., 2006). Thus, we suggest that future research investigates whether the overall lack of differences between the different emotional faces in the present study, particularly in the low load conditions, is maintained when the face stimuli are presented foveally. Moreover, and given the role of motivation in attentional control deficits in anxiety (Berggren and Derakshan, 2013), additional studies should also investigate if this factor could have interacted with the perceptual load manipulations.

The present study provides a further insight in the understanding of social anxiety by examining how perceptual load impairs attentional control to distractor emotional faces in social anxious individuals. The ability to control the focus of attention is an evolutionary relevant behavior (Gilboa-Schechtman and Shachar-Lavie, 2013), as well as an important social tool for social interaction regulation (Gilbert et al., 2009) and should, therefore, deserve further research.

## Author Contributions

SS, CS, TN designed the experiment; PR programmed the experiment; TN and MR collected the data under the supervision of SS and CS; MF and TN analyzed the data in collaboration with SS and CS; SS wrote the manuscript.

### Conflict of Interest Statement

The authors declare that the research was conducted in the absence of any commercial or financial relationships that could be construed as a potential conflict of interest.
